# COVID-19 Testing and Case Rates and Social Contact Among Residential College Students in Connecticut During the 2020-2021 Academic Year

**DOI:** 10.1001/jamanetworkopen.2021.40602

**Published:** 2021-12-23

**Authors:** Olivia Schultes, Victoria Clarke, A. David Paltiel, Matthew Cartter, Lynn Sosa, Forrest W. Crawford

**Affiliations:** 1 Yale School of Public Health, New Haven, Connecticut; 2 Connecticut Department of Public Health, Hartford

## Abstract

**Importance:**

During the 2020-2021 academic year, many institutions of higher education reopened to residential students while pursuing strategies to mitigate the risk of SARS-CoV-2 transmission on campus. Reopening guidance emphasized polymerase chain reaction or antigen testing for residential students and social distancing measures to reduce the frequency of close interpersonal contact, and Connecticut colleges and universities used a variety of approaches to reopen campuses to residential students.

**Objective:**

To characterize institutional reopening strategies and COVID-19 outcomes in 18 residential college and university campuses across Connecticut.

**Design, Setting, and Participants:**

This retrospective cohort study used data on COVID-19 testing and cases and social contact from 18 college and university campuses in Connecticut that had residential students during the 2020-2021 academic year.

**Exposures:**

Tests for COVID-19 performed per week per residential student.

**Main Outcomes and Measures:**

Cases per week per residential student and mean (95% CI) social contact per week per residential student.

**Results:**

Between 235 and 4603 residential students attended the fall semester across each of 18 institutions of higher education in Connecticut, with fewer residential students at most institutions during the spring semester. In census block groups containing residence halls, the fall student move-in resulted in a 475% (95% CI, 373%-606%) increase in mean contact, and the spring move-in resulted in a 561% (95% CI, 441%-713%) increase in mean contact compared with the 7 weeks prior to move-in. The association between test frequency and case rate per residential student was complex; institutions that tested students infrequently detected few cases but failed to blunt transmission, whereas institutions that tested students more frequently detected more cases and prevented further spread. In fall 2020, each additional test per student per week was associated with a decrease of 0.0014 cases per student per week (95% CI, –0.0028 to –0.00001).

**Conclusions and Relevance:**

The findings of this cohort study suggest that, in the era of available vaccinations and highly transmissible SARS-CoV-2 variants, colleges and universities should continue to test residential students and use mitigation strategies to control on-campus COVID-19 cases.

## Key Points

**Question:**

What is the association between COVID-19 testing and case rates on residential college campuses?

**Findings:**

In this cohort study of 18 Connecticut colleges and universities, infrequent COVID-19 testing of residential students was not associated with decreased transmission, whereas testing of residential students twice per week was associated with decreased transmission during the 2020-2021 academic year.

**Meaning:**

Findings suggest that twice-weekly COVID-19 testing of residential students may serve as an effective infection mitigation strategy at colleges and universities.

## Teaser

This cohort study assesses the associations between COVID-19 testing and case rates and social contact to characterize reopening strategies for residential students at college and university campuses in Connecticut.

## Introduction

Institutions of higher education throughout the United States ended in-person education in response to the COVID-19 pandemic in the spring of 2020.^1^ During the summer of 2020, policy makers, advocates, researchers, and university leaders developed guidelines and policies for safely reopening colleges and universities to residential students and in-person education.^2,3,4,5,6,7^ A major point of controversy was the importance of viral testing for residential students. The Centers for Disease Control and Prevention (CDC) published guidance that did not recommend SARS-CoV-2 viral testing on arrival on campus.^8,9^ Other guidelines published during the summer of 2020 did not specify a recommended frequency for testing of residential students or staff.^4,6^ By October 2020, the CDC still had not explicitly recommended either entry screening or routine asymptomatic testing.^10^ Researchers developed mathematical models of disease transmission to estimate campus COVID-19 outcomes,^11,12,13,14,15^ providing similarly varied prescriptions for safe reopening: some recommended testing only a fraction of students per week,^16,17^ whereas others recommended at least twice-weekly screening.^11,12^ Late in the summer of 2020, researchers warned^18^ about the risk of significant outbreaks resulting from low testing rates^14,19^ and inequalities in access to comprehensive testing.^20^

In Connecticut, where about 190 000 students are enrolled in institutions of higher education,^2^ Governor Ned Lamont closed public schools on March 17, 2020, and encouraged private schools to close.^21^ In early summer 2020, the education committee of the Reopen Connecticut Advisory Group recommended viral testing as a central part of institutions’ reopening plans.^2^ Each residential institution of higher education in Connecticut was required to submit a reopening plan to the Connecticut Department of Public Health (CT DPH) addressing repopulation, monitoring, case containment, and campus shutdown.^22^ Throughout summer 2020, the education committee issued additional guidance for fall 2020 in consultation with the CT DPH,^3^ including a 14-day quarantine for residential students arriving from high-incidence states,^7^ testing on arrival or documented testing before arrival,^3^ and viral testing of 5% to 10% of residential students per week throughout the fall 2020 semester.^3^ Those recommended testing rates fell far below the levels separately recommended by transmission modeling analyses for suppression of outbreaks.^11,12^ The anticipated costs of residential screening programs may have influenced the CT DPH testing frequency recommendations.^5,23^

Despite conflicting recommendations and guidelines, most institutions of higher education in Connecticut reopened in fall 2020 and continued in-person residential education through spring 2021. Updated guidance for spring was released January 8, 2021,^24^ which recommended testing before arrival to campus, quarantine after arrival to campus, and increased weekly residential testing through the end of February 2021. Updates on February 25 and March 26 extended the weekly testing recommendation through the end of the spring semester.^25,26^ On April 1, 2021, all Connecticut residents 16 years or older became eligible to receive a COVID-19 vaccine, and COVID-19 case counts began to decline statewide.

Reports of campus outbreaks and other experiences have informed institutions’ plans for fall 2021,^27,28^ but uncertainty remains about the changing risk environment due to increased levels of immunity through natural infection and vaccination as well as to the threat posed by new variants. Although effective vaccines are available in the US, vaccination rates among students may not be sufficient to prevent outbreaks on campus. Assembling and evaluating the limited evidence about what did or did not work would contribute to prevention portfolio design and planning for the safe return to normalcy in higher education. In this study, we explored the associations between observed social contact, testing rate, and case rate at 18 residential college campuses in Connecticut. We evaluated the hypothesis that institutions with lower COVID-19 testing rates would have higher COVID-19 case rates.

## Methods

This cohort study study followed the Strengthening the Reporting of Observational Studies in Epidemiology (STROBE) reporting guideline. This work was approved as not human subjects research by the Yale University institutional review board.

We studied 18 Connecticut institutions of higher education that opened for at least partial in-person instruction and housed residential students during the fall 2020 semester. For institutions with more than 1 campus, we studied only the main campus housing residential students. Institutions included the main campus of the University of Connecticut, the 4 state universities (Central, Eastern, Southern, and Western Connecticut State Universities), and 13 private universities (Albertus Magnus College, Connecticut College, Fairfield University, Mitchell College, Quinnipiac University, Sacred Heart University, Trinity College, University of Bridgeport, University of Hartford, University of New Haven, University of Saint Joseph, Wesleyan University, and Yale University). A map of the included 18 universities is provided in eFigure 1 in the Supplement.

The University of Connecticut, Connecticut State Colleges & Universities (CSCU), and the Connecticut Conference of Independent Colleges provided data on residential enrollment and move-in dates. Of 18 colleges and universities, we obtained official reopening plans for at least 1 semester from all schools except Connecticut College, whose reopening plans we obtained from publicly available data. Additional data were collected from publicly available documents, including the 2019 Connecticut Higher Education System Data and Trends Report.^29^

Universities reported the number of COVID-19 tests conducted each week and the number of positive tests (cases) recorded. Case rates include positive tests from university screening programs and student-reported test results. Residential testing and case data from the University of Connecticut were obtained from university officials and a public dashboard.^30^ The CSCU data included only polymerase chain reaction tests with no pooling. The University of Connecticut and Connecticut Conference of Independent Colleges member institutions reported both polymerase chain reaction and antigen tests, and the University of Hartford reported pooled testing (with each individual tested reported as a single test). Some data were incomplete: CSCU collected only residential testing data starting September 20, 2020; commuter testing data starting October 4, 2020; and self-reported cases (both residential and commuter) starting March 14, 2021. From August 24 to October 5, 2020, Yale University reported residential testing rates more than twice the values reported in other weeks. Those rates may represent combined residential and commuter tests because reporting had just begun; data from those weeks were excluded.

We estimated the frequency of close interpersonal contact occurring on and near institutional campuses in Connecticut using mobile device data. The method uses location data from a cohort of mobile devices to detect likely proximity between devices within the CDC-defined 6-foot contact radius,^31,32,33^ aggregated by census block group (CBG).^34^ Contact counts are scaled by the estimated fraction of the total population present in the mobile device cohort based on data from the American Community Survey.^35^ The metric excludes contact occurring in the primary dwell locations of devices (where the device is located most of the time during the prior month) and roadways. Campus-associated contact was defined as contact occurring in the CBG that most overlapped with the portion of campus containing residence halls. Contact occurring off campus but within university-associated CBGs is included in this analysis. We illustrated contact per residential student for comparability across campuses; in statistical regression results, we use unnormalized estimates of total contact.

From the CT DPH, we obtained weekly counts of confirmed COVID-19 cases in Connecticut towns. The 2019 American Community Survey population data^36^ were used to calculate weekly case rates per resident; college students living on or near campus are included in official census counts and estimates.^37^ Weekly residential testing, case, and contact rates per student per week were calculated for each institution for the fall 2020 and spring 2021 semesters. At most institutions, fall semester took place from August or September to December, and spring semester took place from January or February to May or June. For contact, test, and case rates, we defined the on-campus academic period as the week of moving in through the week of moving out.

### Statistical Analysis

Statistical analyses were conducted using R, version 4.0.4 (R Project for Statistical Computing).^38^ We used linear regression to estimate associations between institutional testing rates and cases per student by week and by semester. These associations are adjusted for the town case rate during the same time interval. We also used linear regression to estimate the change in the rate of close interpersonal contact when residential students were on campus compared with the 7 weeks prior to student arrival. This analysis is stratified by semester. All regression analyses report coefficient estimates and 95% CIs. Additional information about statistical models is presented in the eMethods in the Supplement.

## Results

### University Plans and Policies

The 18 institutions included in this study brought back between 235 and 4603 residential students in the fall of 2020, and most institutions had fewer residential students in the spring 2021 semester (eTable 1 in the Supplement). Prepandemic residential housing numbers were not available, although some schools reported a decreasing overall number of on-campus students (Eastern Connecticut State University, University of Bridgeport, and Yale University)^39,40,41^ or limiting rooms to 2 students (Connecticut College, Quinnipiac University, Southern Connecticut State University, University of Connecticut, and Wesleyan University).^42,43,44,45,46^ To dedensify the residential population, each of the 18 colleges and universities offered a mix of in-person and remote learning options. All institutions followed the CT DPH isolation and quarantine guidelines and reserved part of their residential space to accommodate on-campus students.^47^ In addition, all institutions conducted contact tracing as required by the CT DPH (eAppendix in the Supplement).^47^

The 18 institutions and their planned testing policies prior to the fall 2020 and spring 2021 semesters, according to public documents and plans submitted to the CT DPH, are given in eTable 1 in the Supplement. In the fall, 10 institutions required that students test negative for COVID-19 within 14 days of returning to campus, in line with state recommendations.^3^ Other institutions adopted stricter policies, with 7 institutions requiring tests within 7 days of moving in and 4 institutions testing students on arrival to campus. Most institutions included plans to comply with an initial 14-day quarantine for students coming from “hot spot” areas as mandated by Executive Order No. 7III.^7,48^ Some institutions required or recommended quarantine for all students prior to or at the time of arrival on campus. All institutions planned on testing at least 5% to 10% of residential students every week as recommended by the education committee and the CT DPH,^3^ whereas Quinnipiac University stated it would test 15% per week. Connecticut College, Trinity College, Wesleyan University, and Yale University committed to testing all residential students at least once per week.

The CT DPH issued updated guidance for spring 2021 in early January, recommending entry testing, an onboarding quarantine of 7 to 14 days, and weekly testing for all residential students.^24^ In accordance with updated recommendations, most institutions planned to test all residential students at least once weekly through the end of February. The CT DPH weekly testing recommendations were later extended through the end of the spring semester. Most schools adopted a daily health check for those living or working on campus during the fall and spring semesters.

### COVID-19 Testing of Residential Students

**Figure 1.**
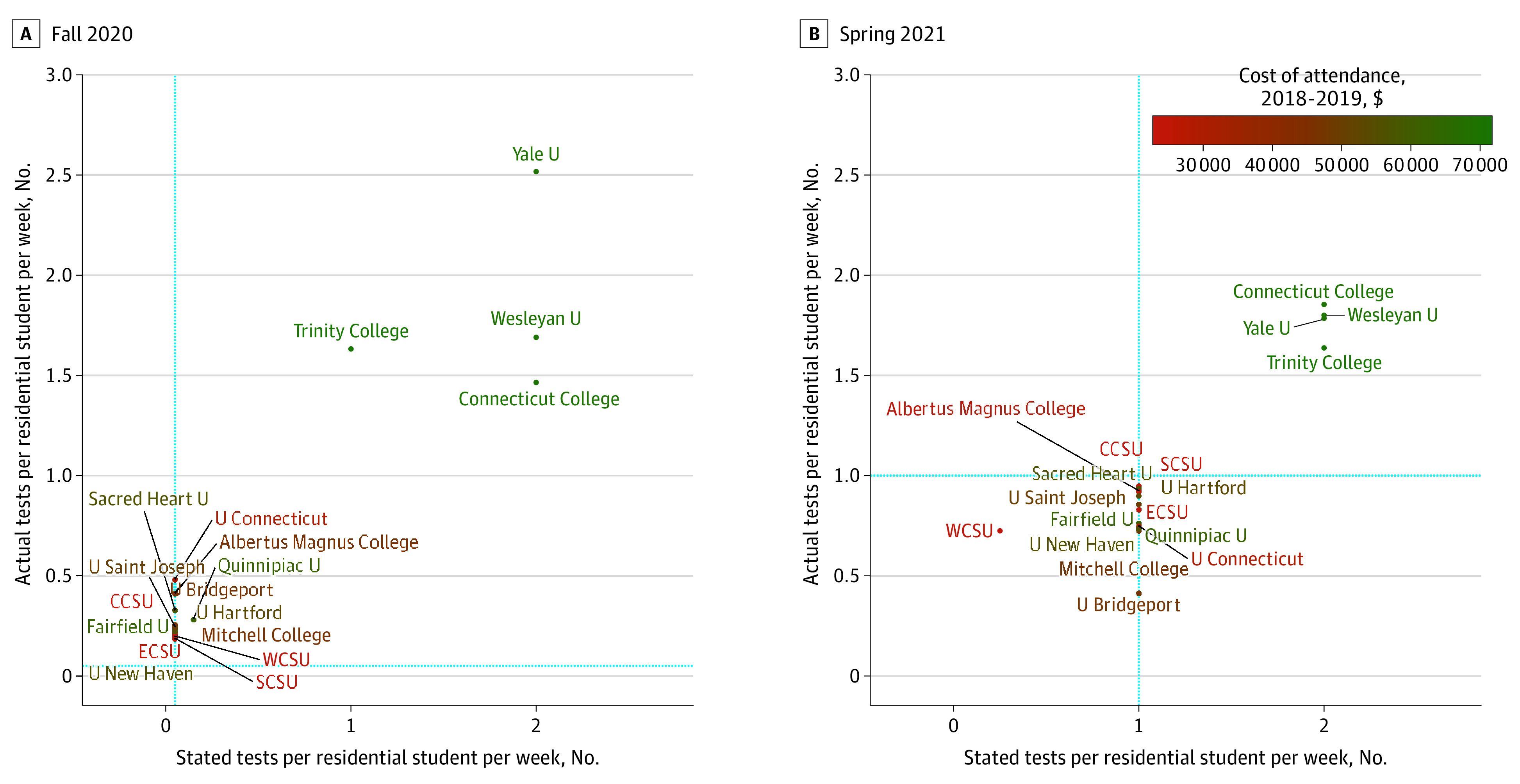
Planned and Actual Residential Student Testing Rates by Semester CCSU indicates Central Connecticut State University; ECSU, Eastern Connecticut State University; SCSU, Southern Connecticut State University; U, University; and WCSU, Western Connecticut State University. The blue dotted lines indicate Connecticut Department of Public Health–recommended testing rates for each semester.

Figure 1 compares planned and actual COVID-19 tests per student for each institution during fall 2020 and spring 2021 as well as the annual cost of attendance.^29^ During fall 2020, all institutions met or exceeded the recommended threshold of testing at least 5% of residential students for COVID-19 on average per week,^3^ but testing rates fell short of planned and recommended thresholds during spring 2021.^24,25,26^ Actual COVID-19 tests performed per residential student per week ranged from 0.18 to 2.08 in fall 2020 and from 0.38 to 1.58 in spring 2021. Connecticut College, Trinity College, Wesleyan University, and Yale University reported testing students on average more than once per week in both semesters.

**Figure 2.**
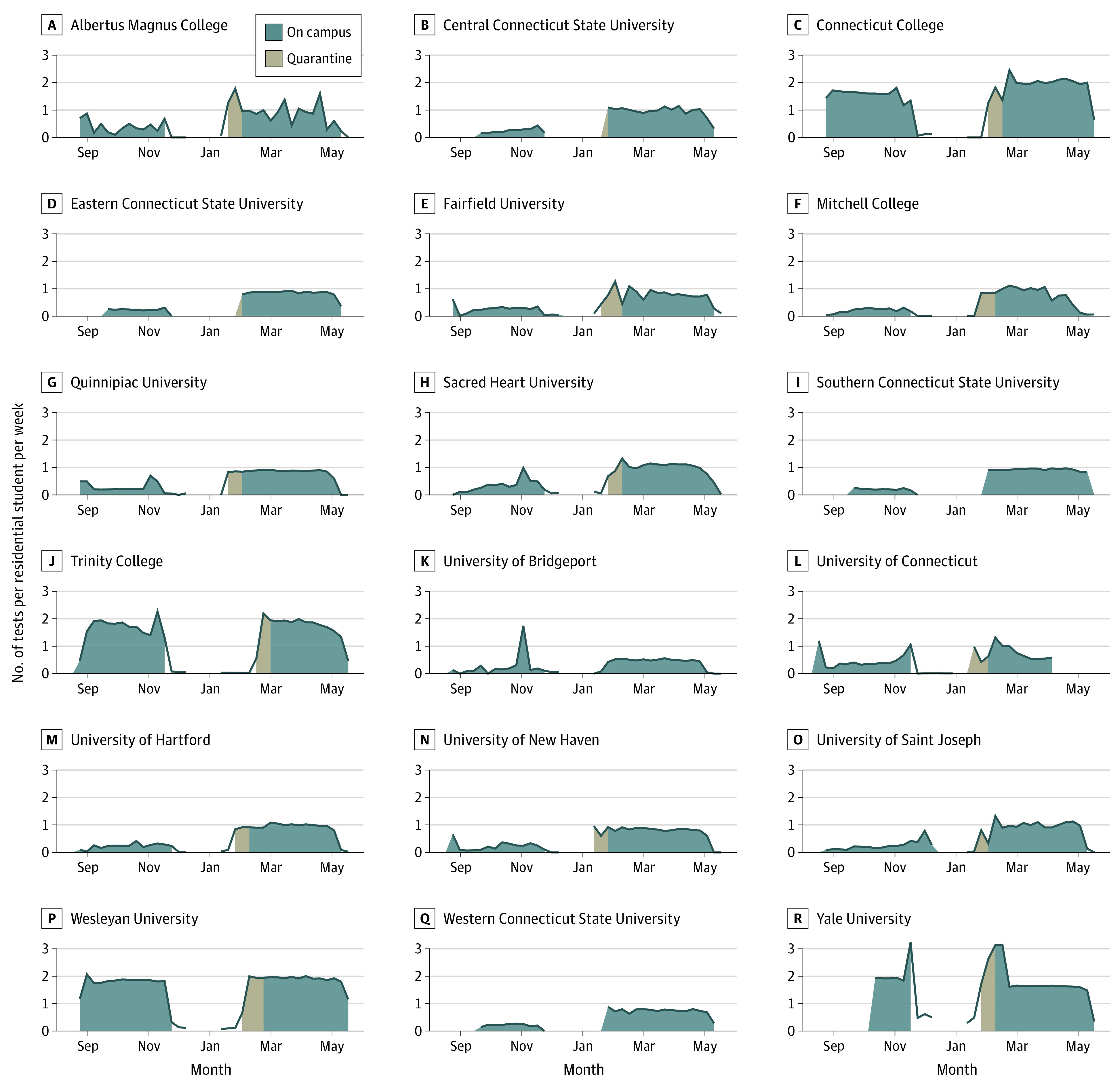
Number of COVID-19 Tests per Residential Student per Week

Figure 2 shows COVID-19 testing rates per residential student during fall 2020 and spring 2021. Testing was relatively constant throughout the fall semester, although some schools conducted increased testing on the move-in date, and several expanded testing during outbreaks. During spring 2021, testing was again mostly constant throughout the semester with the exception of Albertus Magnus College, which increased testing volume in response to several outbreaks throughout spring 2021. Four institutions increased testing during the initial quarantine period. Most other institutions tested at least 75% of their students weekly during spring. Across both semesters, only 4 institutions consistently tested students roughly twice per week.

### Close Interpersonal Contact on or Near Campus

**Figure 3.**
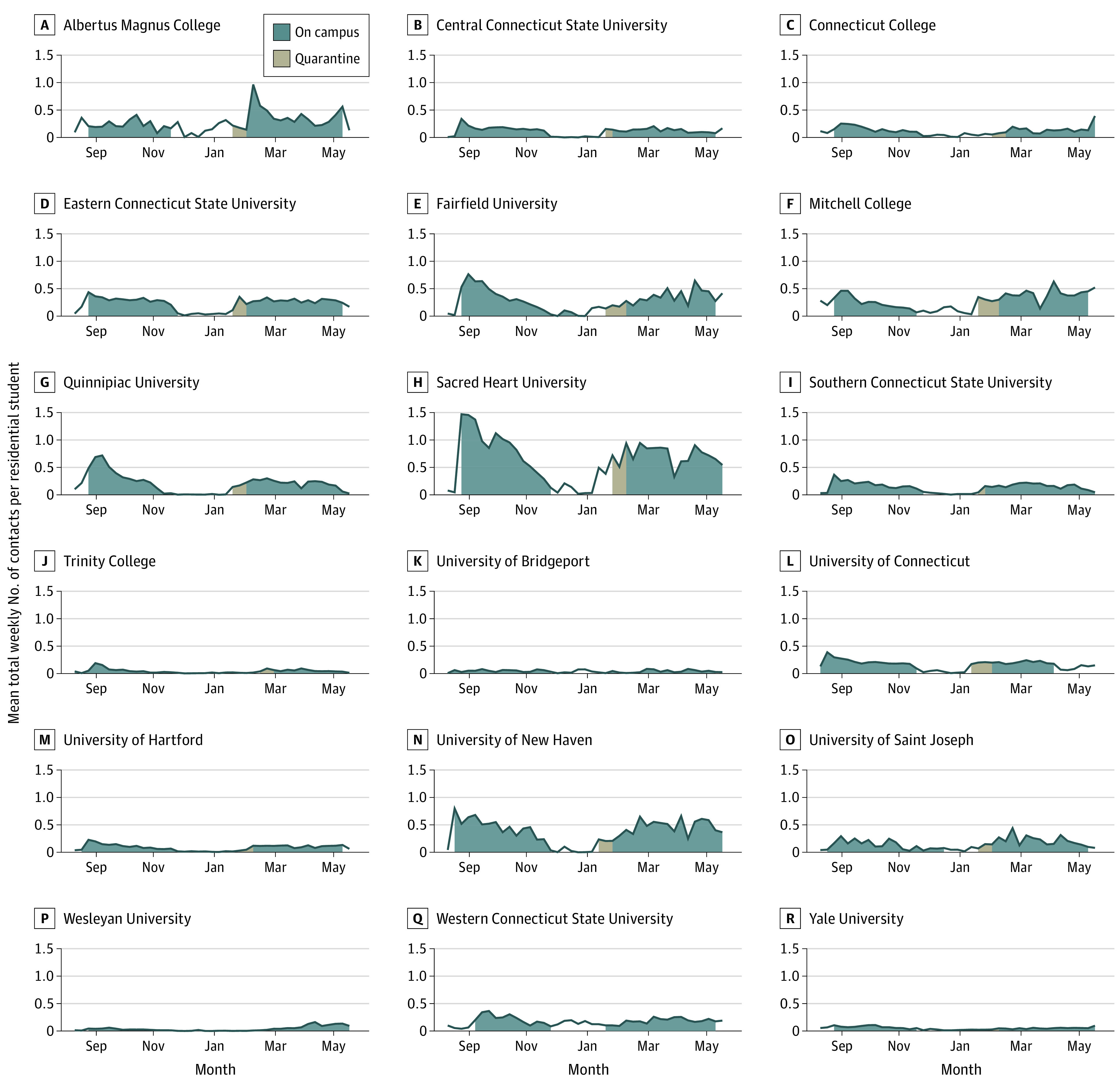
Estimated Number of Close Interpersonal Contact per Residential Student per Week in University-Associated Census Block Groups

Close interpersonal contact increased in university-associated CBGs beginning in late August, with fall 2020 contact per residential student per week peaking at most institutions in early to mid-September. The 4 institutions testing residential students more than once per week had lower rates of contact than other schools. Figure 3 shows the estimated number of close interpersonal contact events per residential student per week in university-associated CBGs.

Contact rates significantly increased during weeks when residential students were on campus compared with the 7 weeks prior to arrival of students: by 475% (95% CI, 373%-606%) in fall 2020 and by 561% (95% CI, 441%-713%) in spring 2021. In other words, the presence of residential students increased close contact rates approximately 5-fold compared with weeks when residential students were not on campus. Contact rates as a function of time before and after the move-in week are shown in eFigures 2 and 3 in the Supplement, and eFigure 4 in the Supplement shows estimates of the log contact rate across institutions as a function of weeks before and after the move-in date. Contact remained high for the first 3 weeks following campus arrival and then slowly decreased throughout the remainder of the semester. During spring 2021, contact increased gradually in the weeks leading up to the move-in date.

### Reported COVID-19 Cases Among Residential Students

**Figure 4.**
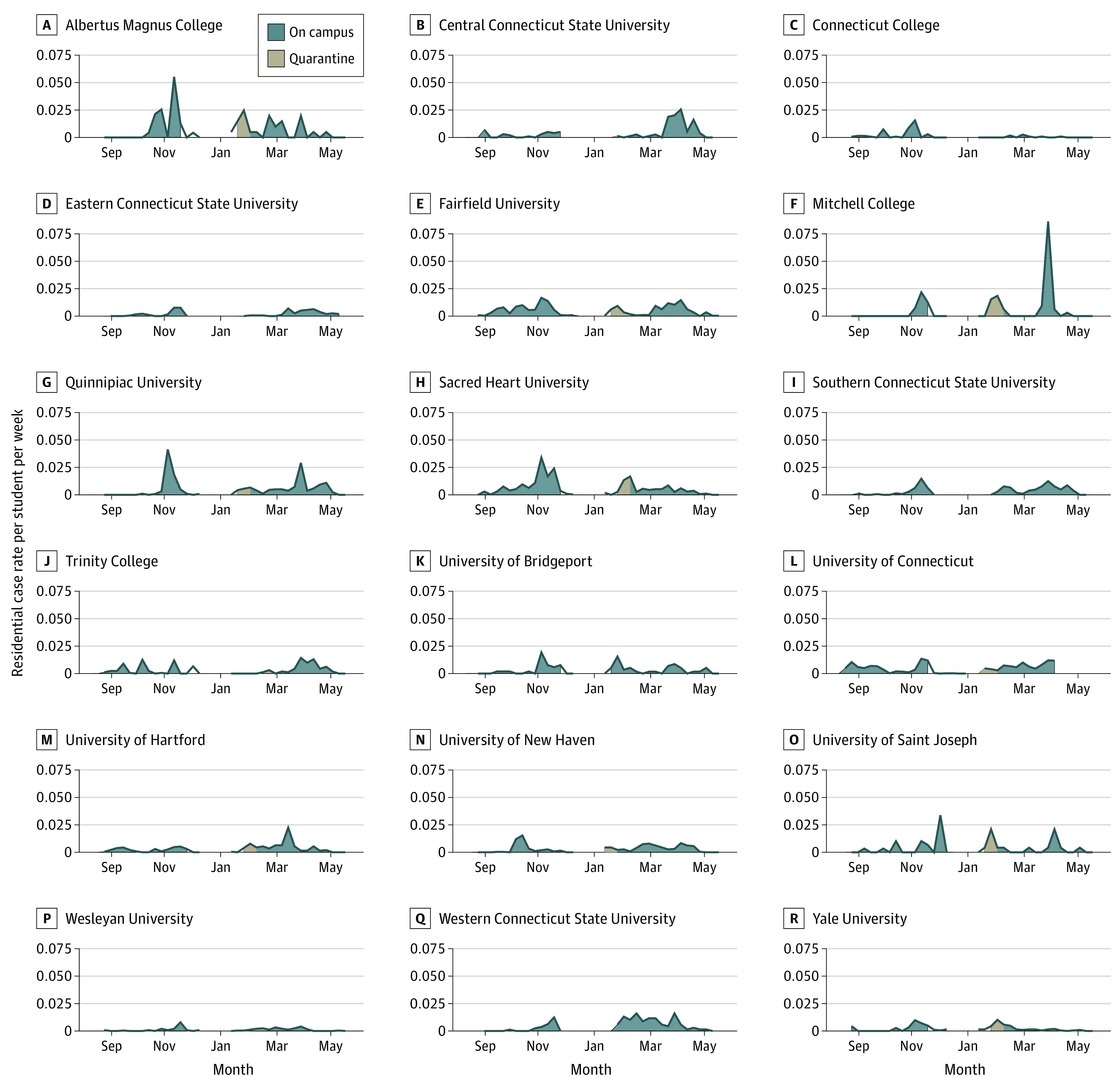
Residential Student Case Rate per Student per Week by Institution

Most institutions reported low residential case rates during the fall semester until early November, when most schools experienced an increase in cases. Figure 4 shows that increases in the per-student case rate typically occurred before exit testing prior to moving out. Reported case patterns were more varied during spring 2021. Panel A of eFigure 5 in the Supplement shows that both aggregated residential case rates and testing rates were higher at most institutions during the spring semester. Institutions with higher testing rates during the entire semester had lower COVID-19 case rates in fall 2020, adjusting for the case rate in the town where the school was located. We fit 2 regression models—1 using data aggregated by week and 1 using data aggregated by semester—to estimate these results and produce the corresponding figures. During the entire semester, there was a modest negative association between testing and cases. Each additional test per student per week was associated with 0.0014 fewer cases per student per week (95% CI, −0.0028 to −0.00001), adjusted for the town case rate (eTable 2 in the Supplement). Panel B of eFigure 5 in the Supplement shows the association between residential student case rates per student per week and per-person case rates in the town where the school was located during the fall and spring semesters. During fall 2020, the overall residential student case rate was positively associated with the town case rate of 0.0845 (95% CI, 0.0607-0.1080), whereas no significant association was observed for spring 2021 (0.0187; 95% CI, −0.0023 to 0.0397). Estimated regression coefficients and confidence intervals are given in eTables 2 and 3 in the Supplement.

**Figure 5.**
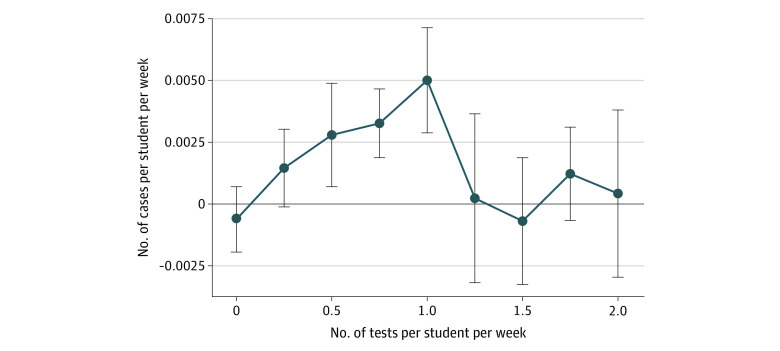
Estimates of Residential Cases per Student per Week by Residential Student Testing Rate Points indicate estimates; bars, 95% CIs.

A weekly analysis of the association between test and reported case rates per residential student revealed a more complex finding. Figure 5 shows that test rates below 2 times per student per week were positively associated with case rates per student, whereas the association for test rates above twice per week was not significantly different from 0 (eTable 4 in the Supplement). The positive association between testing and reported cases was largest for moderate (0.5 to 1.5 tests per student per week) levels of testing and lowest for the most infrequent (between 0 and 0.5 tests per student per week) and most frequent (>2 tests per student per week) testing strategies.

Institutions with higher mean contact per residential student throughout the semester had higher reported case rates, although this association was stronger in the spring (eFigure 6 in the Supplement). During the spring semester, the 4 institutions with the highest residential testing rates also had the lowest reported residential case rates. A plot of the percentage of residential cases detected through the university testing program vs tests per residential student per week is shown in eFigure 7 in the Supplement. A plot of the total number of campus cases vs the number of residential students is shown in eFigure 8 in the Supplement.

## Discussion

Institutions of higher education adopted a variety of approaches to mitigating the risks of reopening residential education during a challenging 2020-2021 academic year. With the availability of effective COVID-19 vaccines in the US, higher education leaders had to design a portfolio of vaccination, testing, contact tracing, and distancing strategies for fall 2021 that would strike an acceptable balance between safety and the return to normalcy. The CDC recommendations for institutions of higher education for fall 2021 emphasized the importance of maximizing vaccination coverage.^49^ As of October 21, 2021, more than 1000 colleges and universities in the US have mandated vaccination for either students or employees, leaving vaccination optional at most institutions.^50^ The emergence of the Delta variant of SARS-CoV-2^51^ highlights the continued need to evaluate and implement infection control measures, such as testing and social distancing. The increased transmissibility of the Delta variant creates more opportunities for vaccine breakthrough cases, increasing the value of testing and other mitigation strategies even in populations with high vaccination rates.^52^ New CDC guidelines for institutions of higher education recommend universal entry screening and asymptomatic screening once or twice per week based on risk in the surrounding community, with priority shifting to unvaccinated persons as vaccine coverage increases.^53^ Guidelines published by the American College Health Association recommend weekly COVID-19 testing for unvaccinated students but do not make a clear general recommendation on testing.^54^

Conflicting guidance related to the frequency of campus testing may have contributed to low COVID-19 testing rates at some institutions, especially during fall 2020. Lack of clarity from CDC guidelines^8^ may have led to uncertainty among policy makers and educational leaders about the importance of testing. In Connecticut, recommendations from the Reopen CT education committee^3^ and CT DPH^24,25,26^ set testing thresholds far below those recommended by transmission modeling studies^11,12^ to prevent campus outbreaks. Those thresholds were also set during early summer 2020 when testing capacity was limited and expensive.^2^ Institutions that met the minimum recommendation during fall 2020 generally experienced higher case rates than those that tested at least once per week. Although frequent testing in residential institutions may be costly, testing differences across institutions were only partly explained by residential tuition and fees.

One of the key findings of this study is the complex association between COVID-19 testing of residential students and recorded residential COVID-19 cases. Testing rates below 0.5 per student per week likely detected few asymptomatic infections; as a result, asymptomatic infectious students were not identified and isolated, enabling unabated spread of prevalent infections. Testing rates above 1.5 per student per week likely detected most infections (including asymptomatic) among students, resulting in isolation of infected individuals and suppression of further transmission. Both scenarios—low and high rates of per-student testing—resulted in low reported case rates. By contrast, moderate testing rates between 0.5 and 1.5 tests per student per week resulted in the highest reported case counts. Moderate testing rates are sufficiently frequent to detect many prevalent infections but not frequent enough to stop most forward transmission and outbreaks. Previous studies have observed that congregate settings with increased testing rates also have increased incidence rates.^55,56,57^ Other research has shown the ability of serial testing to halt outbreaks in these settings.^58^ Our findings contribute to the literature in showing the continuum of this association by comparing settings with low testing rates to those with high testing rates.

Researchers have suggested that SARS-CoV-2 infections on campus caused an increase in community incidence elsewhere.^59,60,61,62^ We observed a positive association during fall 2020 between residential student case rates and case rates in the town where the school was located. However, it is not possible to determine whether on-campus infections were transmitted to the broader community or vice versa. Although some institutions insulated their residential populations from the surrounding area via quarantine, an association in case rates persisted during the fall semester, indicating the possibility of cross-transmission between residential students and community members.

Our analysis of contact rates shows disparities in compliance with social distancing guidelines within and around campuses. Fairfield University and Sacred Heart University showed the highest contact rates in fall 2020. Both institutions experienced COVID-19 outbreaks on campus within weeks of residential students moving in. Close interpersonal contact is a necessary but insufficient condition for COVID-19 transmission to occur. Both prevalent infections and high contact rates are needed to spark an outbreak. Institutions can control prevalent infection through frequent asymptomatic testing and rapid isolation of identified cases, whereas control of contact rates is achieved through social distancing guidelines and limits on gatherings.

### Limitations

This study had several limitations. First, we focused on diagnosed COVID-19 cases recorded and reported by universities. Cases may be a poor proxy for infections in institutions where testing rates were low or where asymptomatic testing was infrequent. Second, we used measures of close interpersonal contact in university-associated CBGs as a proxy for COVID-19 transmission risk. Measured contact may include contact occurring off campus among nonstudents. This sample may not be representative of residential students in university-associated CBGs or may fail to detect contact events that resulted in COVID-19 transmission. Third, transmission on campus is influenced by a broad range of factors, including behavior of faculty and staff as well as nonresidential students on campus. Fourth, Connecticut residents 16 years or older became eligible for vaccination on April 1, 2021, but we do not have information on vaccination rates on Connecticut campuses. It is likely that rising vaccination rates on campus mitigated transmission during the remaining weeks of spring 2021.

## Conclusions

Connecticut institutions of higher education implemented varying testing and mitigation strategies for residential students during the 2020-2021 academic year. While institutions with moderate residential student testing rates (once per week) experienced high case rates, those with high testing rates (ie, twice per week) seemed to be able to isolate asymptomatic students in a timely manner, preventing forward transmission. Social contact increased after students moved in during both semesters, and the positive association of residential case rates with town case rates indicate that campuses were not closed communities. In the era of vaccinations and highly transmissible SARS-CoV-2 variants, colleges and universities should continue to test residential students and use mitigation strategies to effectively control on-campus cases.

## Notes

Supplement. **eMethods.** Statistical Methodology **eAppendix.** Additional Results **eFigure 1.** Connecticut Institutions of Higher Education Studied in This Analysis **eFigure 2.** Estimated Frequency of Close Interpersonal Contact per Residential Student in University-Associated Census Block Groups From Move-In Week (Week 0), Fall 2020 **eFigure 3.** Estimated Frequency of Close Interpersonal Contact per Residential Student in University-Associated Census Block Groups From Move-In Week (Week 0), Spring 2021 **eFigure 4.** Estimated Contact per Residential Student per Week Since Move-In Across Institutions **eFigure 5.** (A) Association Between Institutional Test Rate per Residential Student and Case Rate per Residential Student; (B) Association Between Case Rate per Residential Student and Case Rate per Person in the Town Where the School Was Located **eFigure 6.** Cases per Residential Student as a Function of Contact per Residential Student **eFigure 7.** Residential Student Testing Rate and Percent of Residential Cases Detected Through University Testing Program, by Semester **eFigure 8.** Residential Students and Total Campus Cases Among Private Universities, by Semester **eTable 1.** University Characteristics, Residential Enrollment, and COVID-19 Plans and Policies for Residential Students During the 2020-2021 Academic Year **eTable 2.** Linear Regression Estimates and 95% Confidence Intervals Describing the Negative Association Between Residential Test Rate (per Student per Week) and Residential Case Rate (per Student per Week), Adjusted for Town Case Rate per Person **eTable 3.** Linear Regression Estimates and 95% Confidence Intervals Describing the Association Between Residential Case Rate (per Student per Week) and Town Case Rate (per Person per Week) **eTable 4.** Association Between Tests per Student per Week and Cases per Student per Week, by Test Frequency
eReferences.
