# Supplementary material for: COVID-19 Testing and Case Rates and Social Contact Among Residential College Students in Connecticut During the 2020-2021 Academic Year
Source: JAMA Netw Open. 2021 Dec 23;4(12):e2140602. doi: 10.1001/jamanetworkopen.2021.40602 (PMC8703252; doi:10.1001/jamanetworkopen.2021.40602)
Supplement: Supplement. — eMethods. Statistical Methodology eAppendix. Additional Results eFigure 1. Connecticut Institutions of Higher Education Studied in This Analysis eFigure 2. Estimated Frequency of Close Interpersonal Contact per Residential Student in University-Associated Census Block Groups From Move-In Week (Week 0), Fall 2020 eFigure 3. Estimated Frequency of Close Interpersonal Contact per Residential Student in University-Associated Census Block Groups From Move-In Week (Week 0), Spring 2021 eFigure 4. Estimated Contact per Residential Student per Week Since Move-In Across Institutions eFigure 5. (A) Association Between Institutional Test Rate per Residential Student and Case Rate per Residential Student; (B) Association Between Case Rate per Residential Student and Case Rate per Person in the Town Where the School Was Located eFigure 6. Cases per Residential Student as a Function of Contact per Residential Student eFigure 7. Residential Student Testing Rate and Percent of Residential Cases Detected Through University Testing Program, by Semester eFigure 8. Residential Students and Total Campus Cases Among Private Universities, by Semester eTable 1. University Characteristics, Residential Enrollment, and COVID-19 Plans and Policies for Residential Students During the 2020-2021 Academic Year eTable 2. Linear Regression Estimates and 95% Confidence Intervals Describing the Negative Association Between Residential Test Rate (per Student per Week) and Residential Case Rate (per Student per Week), Adjusted for Town Case Rate per Person eTable 3. Linear Regression Estimates and 95% Confidence Intervals Describing the Association Between Residential Case Rate (per Student per Week) and Town Case Rate (per Person per Week) eTable 4. Association Between Tests per Student per Week and Cases per Student per Week, by Test Frequency eReferences. [file jamanetwopen-e2140602-s001.pdf]

Supplementary Online Content

Schultes O, Clarke V, Paltiel AD, Cartter M, Sosa L, Crawford FW. COVID-19 testing and case rates and social contact among residential college students in Connecticut during the 2020-2021 academic year. *JAMA Netw Open*. 2021;4(12): e2140602. doi:10.1001/jamanetworkopen.2021.40602

eMethods. Statistical Methodology
eAppendix. Additional Results
eFigure 1. Connecticut Institutions of Higher Education Studied in This Analysis
eFigure 2. Estimated Frequency of Close Interpersonal Contact per Residential Student in University-Associated Census Block Groups From Move-In Week (Week 0), Fall 2020
eFigure 3. Estimated Frequency of Close Interpersonal Contact per Residential Student in University-Associated Census Block Groups From Move-In Week (Week 0), Spring 2021
eFigure 4. Estimated Contact per Residential Student per Week Since Move-In Across Institutions
eFigure 5. (A) Association Between Institutional Test Rate per Residential Student and Case Rate per Residential Student; (B) Association Between Case Rate per Residential Student and Case Rate per Person in the Town Where the School Was Located
eFigure 6. Cases per Residential Student as a Function of Contact per Residential Student
eFigure 7. Residential Student Testing Rate and Percent of Residential Cases Detected Through University Testing Program, by Semester
eFigure 8. Residential Students and Total Campus Cases Among Private Universities, by Semester
eTable 1. University Characteristics, Residential Enrollment, and COVID-19 Plans and Policies for Residential Students During the 2020-2021 Academic Year
eTable 2. Linear Regression Estimates and 95% Confidence Intervals Describing the Negative Association Between Residential Test Rate (per Student per Week) and Residential Case Rate (per Student per Week), Adjusted for Town Case Rate per Person
eTable 3. Linear Regression Estimates and 95% Confidence Intervals Describing the Association Between Residential Case Rate (per Student per Week) and Town Case Rate (per Person per Week)
eTable 4. Association Between Tests per Student per Week and Cases per Student per Week, by Test Frequency
eReferences.

This supplementary material has been provided by the authors to give readers additional information about their work.

© 2021 Schultes O et al. *JAMA Network Open*.

eMethods.

Statistical methodology

To evaluate the association between the average residential case rate in institution i in semester s with the average test rate in that institution, adjusting for the average case rate in the surrounding town, we fit a linear model

$$(average\ case\ rate)_{is} = \alpha + \beta(average\ test\ rate)_{is} + \gamma(average\ town\ case\ rate)_{is} + \epsilon_{is}$$

where ϵ_{is} is an independent error term for institution i in semester s. The estimated coefficients and 95% confidence intervals are shown in eTable 2.

To evaluate the change in close interpersonal contact when residential students were brought back to campus, we fit the following linear regression model for institution i in week w:

$$(log\ average\ contact)_{iw} = \alpha + \beta(students\ in\ residence)_{iw} + \gamma(semester)_{iw} + \delta(students\ in\ residence)_{iw}(semester)_{iw} + \epsilon_{iw}$$

where the coefficient δ corresponds to the interaction of the indicator that students are in residence and semester, and ϵ_{iw} is an independent error term for institution i in week w. The estimated coefficients and 95% confidence intervals are given in the main text.

To visualize the weekly change in average contact following arrival of residential students on campus, we fit the following model

$$(log\ average\ contact)_{iw} = \beta_w(students\ in\ residence)_{iw}(semester)_{iw} + \epsilon_{iw}$$

where w is measured in weeks relative to arrival of students on campus. eFigure 4 shows estimated weekly coefficients and 95% confidence intervals for both semesters.

To evaluate the association between the residential case rate in institution i in week w with the weekly test rate in that institution, while adjusting for the case rate in the surrounding town, we fit a linear model

$$(case\ rate)_{iw} = \sum_b \beta_b(test\ rate\ in\ bin\ b)_{iw} + \gamma(town\ case\ rate)_{iw} + \epsilon_{iw}$$

where the sum is over discretized weekly residential testing rate with bins (tests per residential student per week):

$$(0, 0.25], (0.25, 0.5], (0.5, 0.75], (0.75, 1.0], (1.0, 1.25], (1.25, 1.5], (1.5, 1.75], (1.75, 2.0], (2.0, \infty)$$

The error term ϵ_{iw} is independent for institution i in week w. This binned model has no intercept so that we can interpret the coefficients β_b as the estimated change in case rate under testing rate in bin b, holding town case rate constant. The estimated coefficients β_b and 95% confidence intervals are shown in Figure 5 in the main text and numerical results are shown in eTable 4.

© 2021 Schultes O et al. *JAMA Network Open*.

To evaluate the association between case rates in towns where institutions are located and case rates in those institutions, we fit a linear model

$$(town\ case\ rate)_{is} = \alpha + \beta_s (campus\ case\ rate)_{is} + \epsilon_{is}$$

where the coefficient β_s corresponds to semester s and ϵ_{is} is an independent error term for institution i in semester s. Estimated coefficients are shown in eTable 3.

eAppendix. Additional Results

Details of university plans and policies

eTable 1 shows summary information on COVID-19 residential student testing policies for 18 Connecticut colleges and universities according to public documents and plans submitted to the Connecticut Department of Public Health (CT DPH). We examined four institutions that grant baccalaureate degrees and eleven that also offer masters or doctoral degrees. The number of residential students in the fall ranged from 235 (Albertus Magnus College) to 4,603 (University of Connecticut). Most institutions had fewer residential students in the spring semester. In the fall, CT DPH recommended that students test negative within 14 days of returning to campus. Ten institutions adopted this policy, while seven required a negative test within 7 days of move-in. The University of Connecticut did not require pre-arrival testing but tested students on move-in day; three other universities also tested students upon move-in. Eleven institutions outlined plans to comply with an initial 14-day quarantine for students coming from "hot spot" areas as mandated by Executive Order 7III[1,2]. Five required all students to quarantine regardless of origin, and two did not have available data on arrival quarantine policies. Thirteen institutions planned for to test 5-10% of residential students each week, the level recommended by CT DPH, while Quinnipiac University stated it would test 15% of students per week. Connecticut College, Trinity College, Wesleyan University, and Yale University stated they would test residential students at least once per week. Twelve institutions implemented a daily health check throughout the semester, while three had a health check for the first 7 days as recommended by CT DPH. Two institutions didn't have include information on a daily health check in their reopening plans.

CT DPH issued updated guidance for spring 2021 in early January, following several college outbreaks during the fall and increasing statewide incidence [3]. Recommendations for residential students included entry testing within 7 days before move-in and on move-in day, a 7-14 day quarantine period, and continued weekly testing through the end of February. Twelve institutions required a test within 7 or fewer days prior to move-in with 5 giving larger windows (i.e., 10 or 14 days) for pre-arrival tests. No data on testing, quarantine policy, or daily health checks was available from the University of Saint Joseph for the spring semester. Eleven school explicitly stated that students were tested on move-in day, while three other schools specified students would be tested within 2-5 days following move-in (Fairfield University), within 6-10 days following move-in (University of Bridgeport) and within 9 days following

© 2021 Schultes O et al. *JAMA Network Open*.

move-in (University of New Haven), respectively. Though no other schools explicitly specified testing students upon arrival, most had plans stating they were conducting weekly testing of residential students at the beginning of the semester; therefore, it is feasible this weekly testing began the day of move-in. Fourteen institutions reported implementing a quarantine of 7 days or longer for residential students, in line with CT DPH recommendations. Four had no available data on initial quarantine. Sixteen institutions planned to test students at least weekly through the end of February, as recommended by CT DPH; as in the fall semester, Connecticut College, Trinity College, Wesleyan University, and Yale University stated plans to test students more than once per week. During the spring semester, CT DPH extended its weekly testing recommendations through the end of the semester[4,5]; this guidance is not reflected in institutions' reopening plans which were submitted prior to the start of the semester. Twelve institutions adopted a daily health check throughout the semester, while three implemented this measure only for the first 7 days following move-in and three did not include information on this in their reopening plans.

Cases as a function of contact

In both the fall and spring semesters, institutions with higher contact rates had higher case rates (eFigure 6). In the fall semester, most colleges were conducting low levels of testing (less than half of residential students per week). The four institutions with the highest testing rates (Connecticut College, Trinity College, Wesleyan University, and Yale University) had low contact rates and low case rates. Case rates were higher in the spring semester, despite higher testing rates and similar or lower testing rates. As in the fall semester, the four institutions testing students twice per week had the lowest contact rates and case rates.

Percent of residential student cases detected through institution screening program

eFigure 7 presents data on the frequency of residential student testing and the proportion of cases detected through each institution's screening program (compared with the proportion self-reported by students). In the fall, Yale University and the four state universities didn't report data on self-reported cases. Of the institutions that reported, four institutions detected nearly all cases through their residential testing program (Connecticut College, Mitchell College, Trinity College, and Wesleyan College); of those institutions, three tested their students more than once per week. The remaining institutions detected at least 50% of residential student cases through the testing program, with the exception of Albertus Magnus College. In the spring, increased testing frequency did not lead to improved proportion of cases detected within the testing program. Three of the four institutions detecting almost every case through the testing program (Connecticut College, Wesleyan University, Western Connecticut State University, and Yale University) tested students more than once per week. It is unclear how practices on recording self-reported tests varied between institutions.

© 2021 Schultes O et al. *JAMA Network Open*.

Cases among residential students and total university-affiliated populations

eFigure 8 examines total campus cases by the number of residential students each semester. In both semesters, institutions with more residential students had more total campus cases. However, among institutions with high absolute number of total cases, the proportion of cases occurring among residential students was not uniform. While high case counts for Quinnipiac University and Fairfield University in the spring semester were driven by a high proportion of residential student cases, these cases only accounted for approximately half of total campus cases in the three schools with the highest case counts in the fall semester (Sacred Heart University, Fairfield University, and Quinnipiac University, respectively).

© 2021 Schultes O et al. *JAMA Network Open*.

eFigure 1. Connecticut Institutions of Higher Education Studied in This Analysis

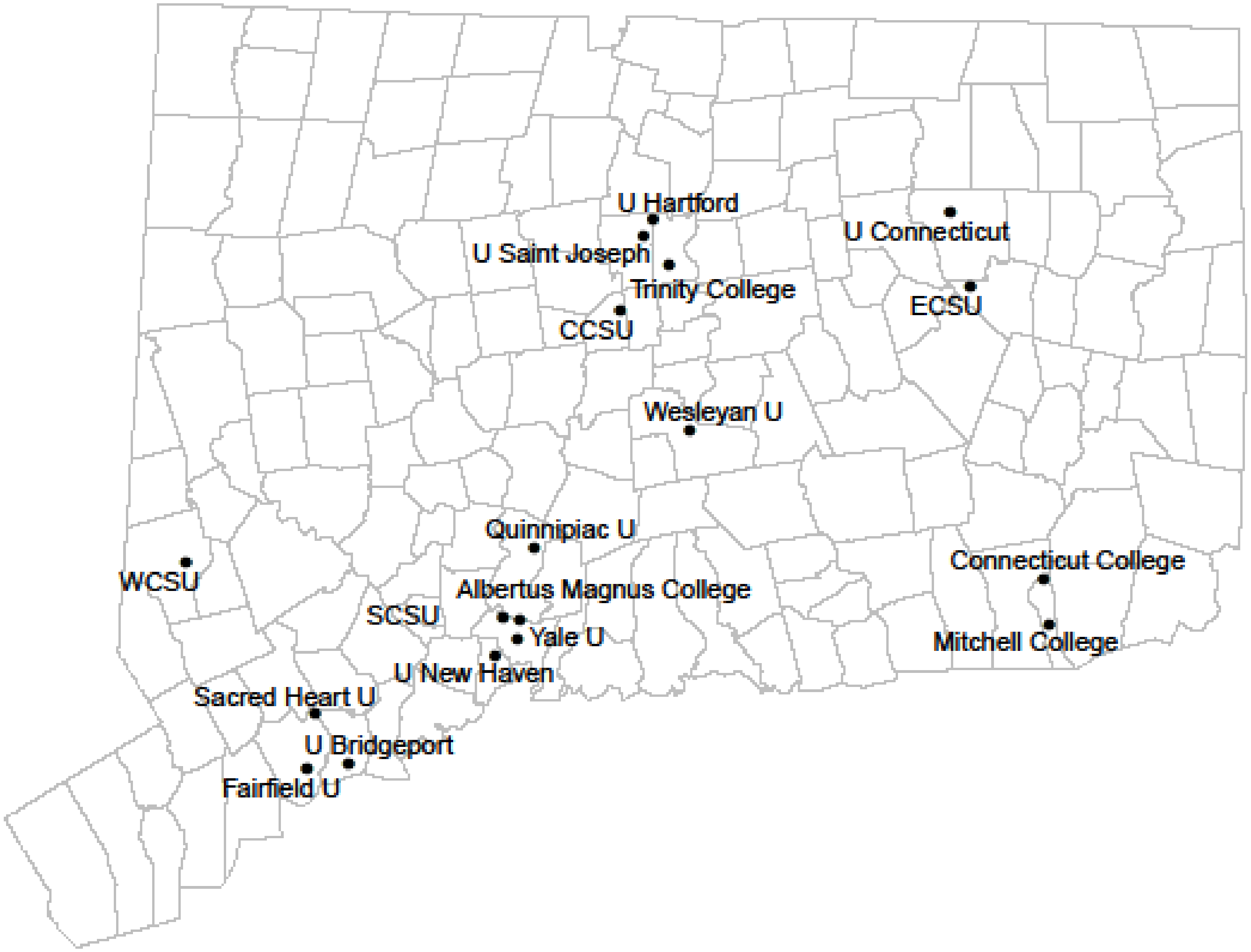

© 2021 Schultes O et al. *JAMA Network Open*.

eFigure 2. Estimated Frequency of Close Interpersonal Contact per Residential Student in University-Associated Census Block Groups From Move-In Week (Week 0), Fall 2020

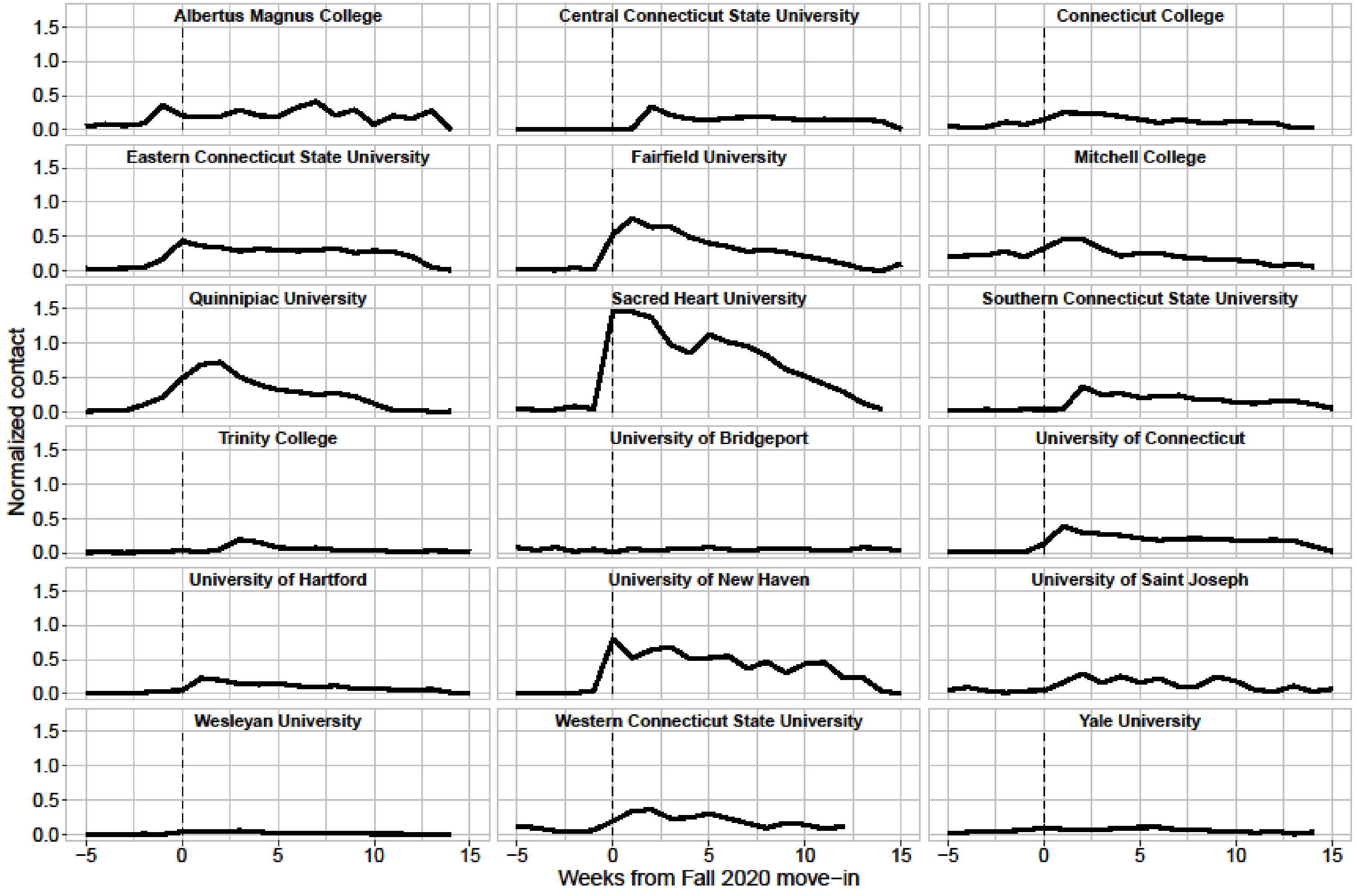

© 2021 Schultes O et al. *JAMA Network Open*.

eFigure 3. Estimated Frequency of Close Interpersonal Contact per Residential Student in University-Associated Census Block Groups From Move-In Week (Week 0), Spring 2021

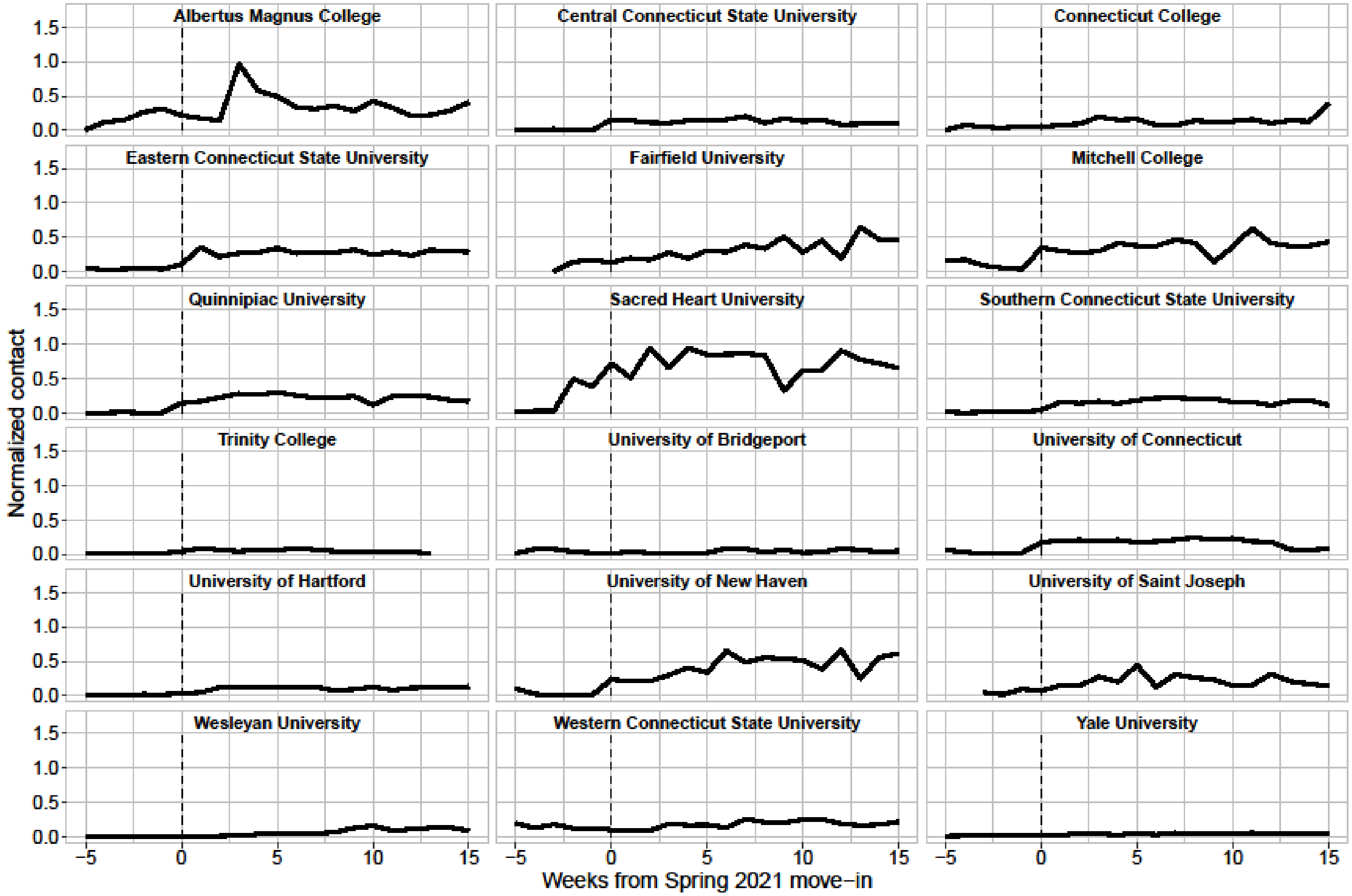

© 2021 Schultes O et al. *JAMA Network Open*.

eFigure 4. Estimated Contact per Residential Student per Week Since Move-In Across Institutions

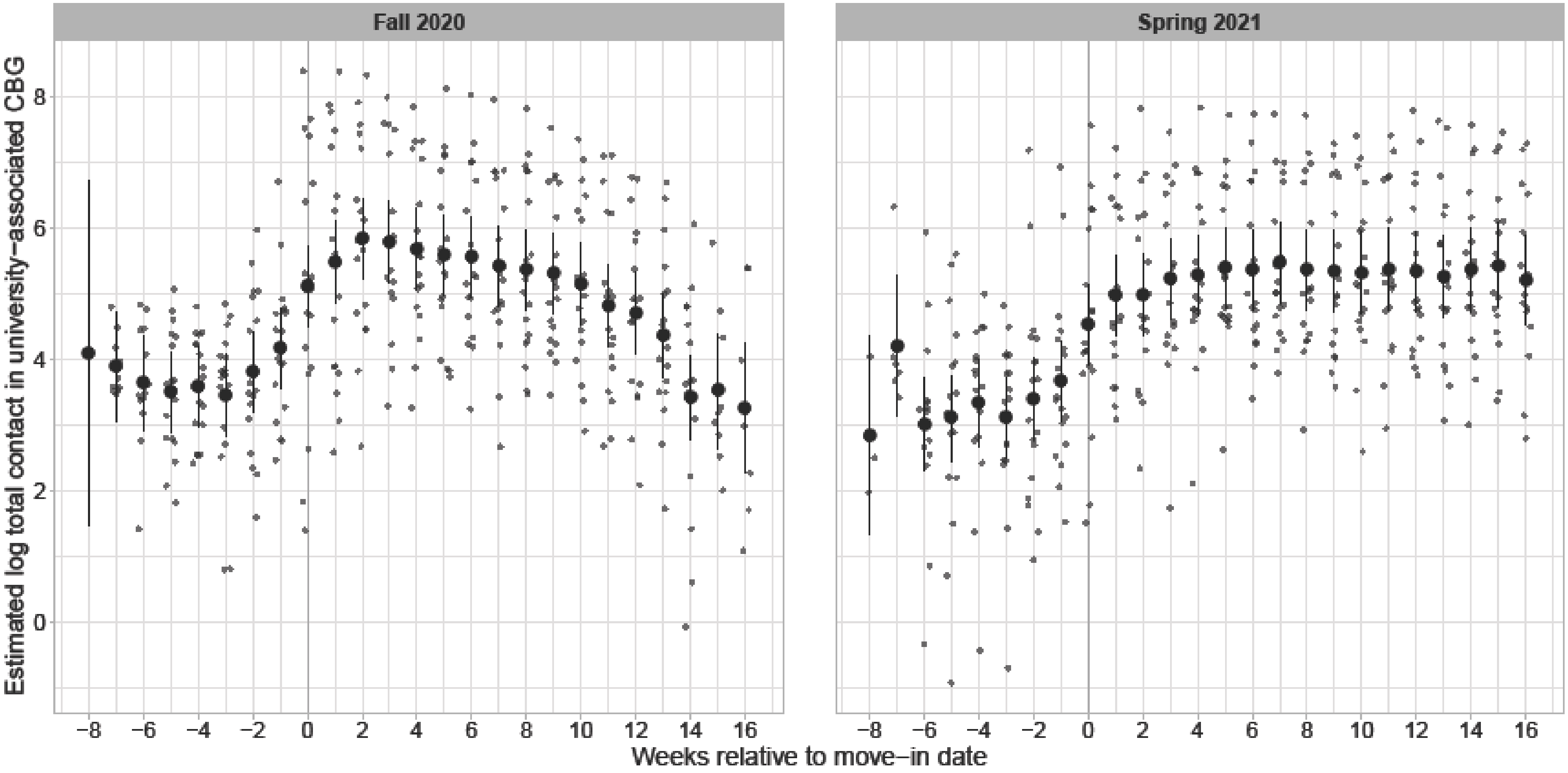

© 2021 Schultes O et al. *JAMA Network Open*.

eFigure 5. (A) Association Between Institutional Test Rate per Residential Student and Case Rate per Residential Student; (B) Association Between Case Rate per Residential Student and Case Rate per Person in the Town Where the School Was Located

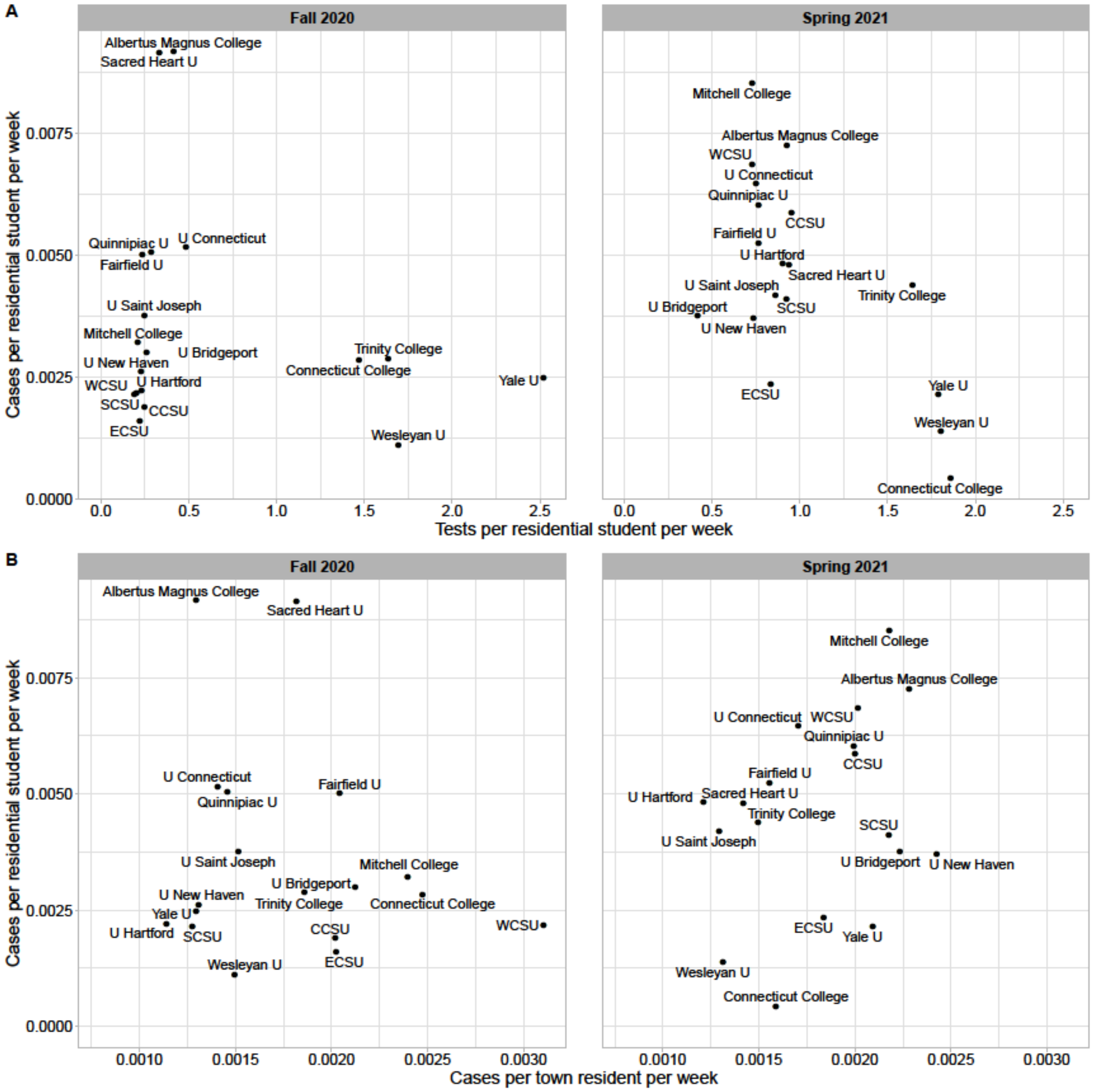

© 2021 Schultes O et al. *JAMA Network Open*.

eFigure 6. Cases per Residential Student as a Function of Contact per Residential Student

Fall 2020 includes data from full weeks between move-in and move-out, while spring 2021 includes data from move-in through the last week of March when college-age individuals became eligible to receive the COVID-19 vaccine in Connecticut.

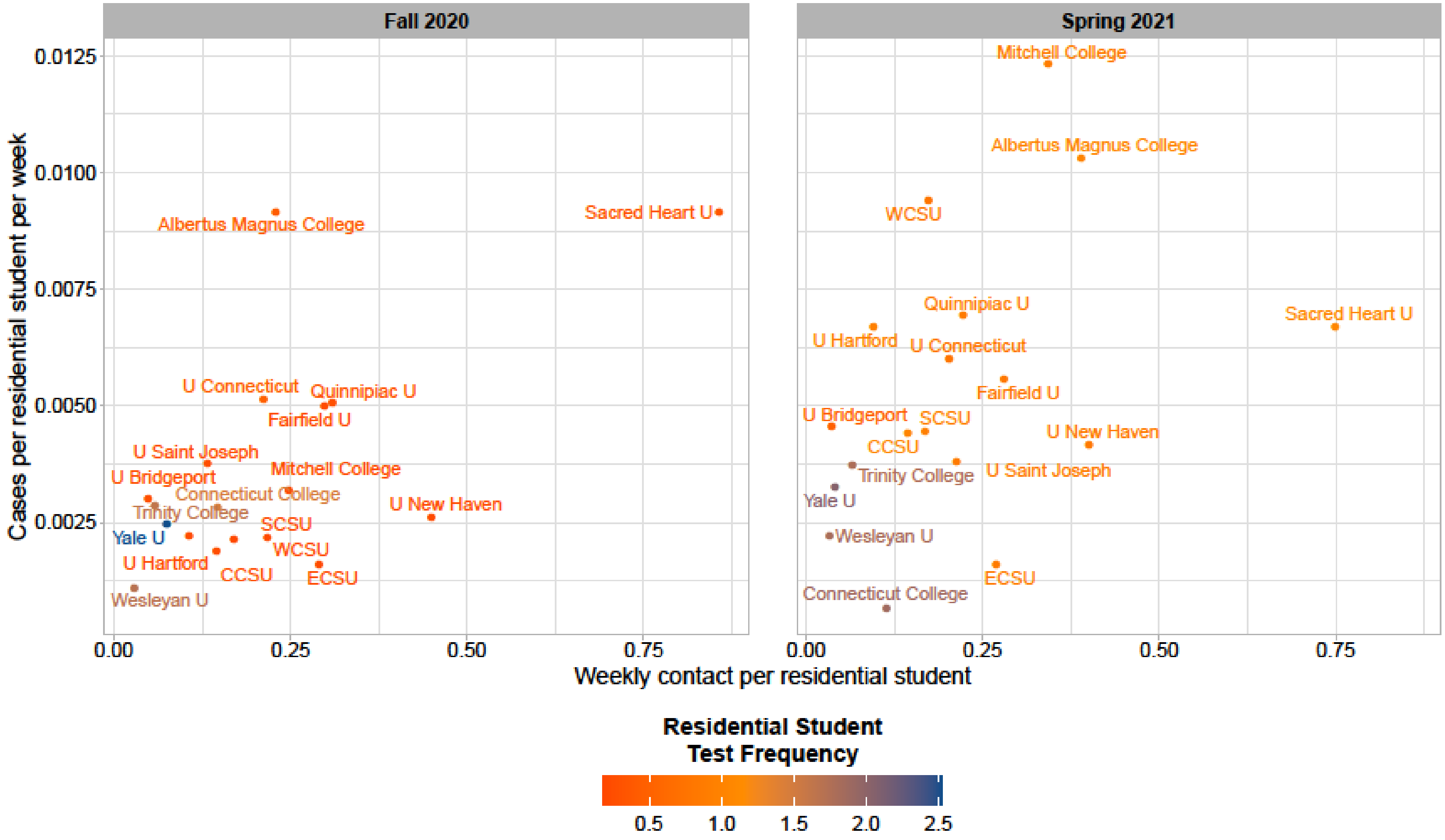

© 2021 Schultes O et al. *JAMA Network Open*.

eFigure 7. Residential Student Testing Rate and Percent of Residential Cases Detected Through University Testing Program, by Semester

Semesters include data from full weeks between move-in and move-out.

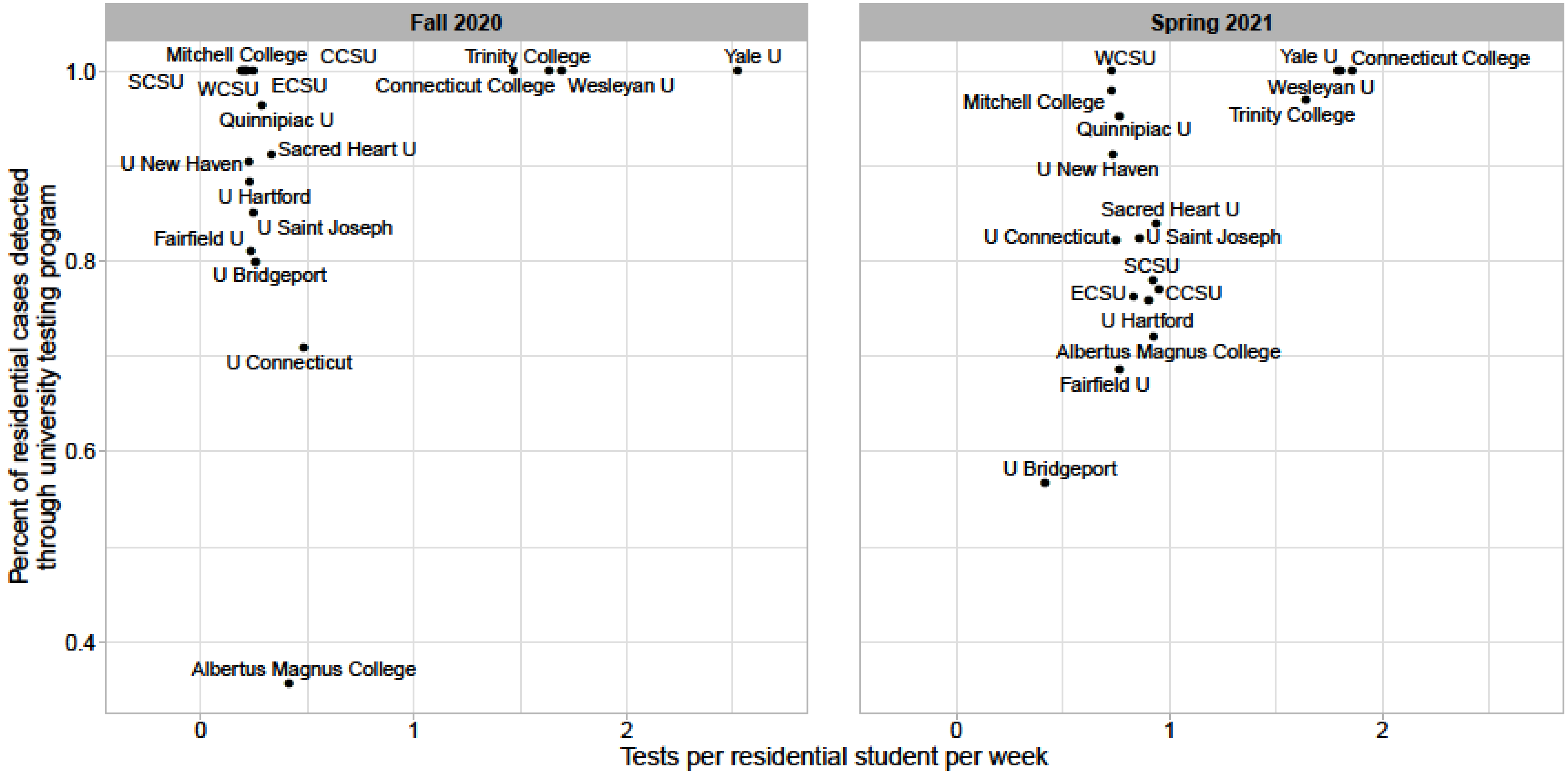

© 2021 Schultes O et al. *JAMA Network Open*.

eFigure 8. Residential Students and Total Campus Cases Among Private Universities, by Semester

Total campus cases include reported cases among residential students, commuter students, and staff/faculty. Semesters include full weeks between move-in and move-out.

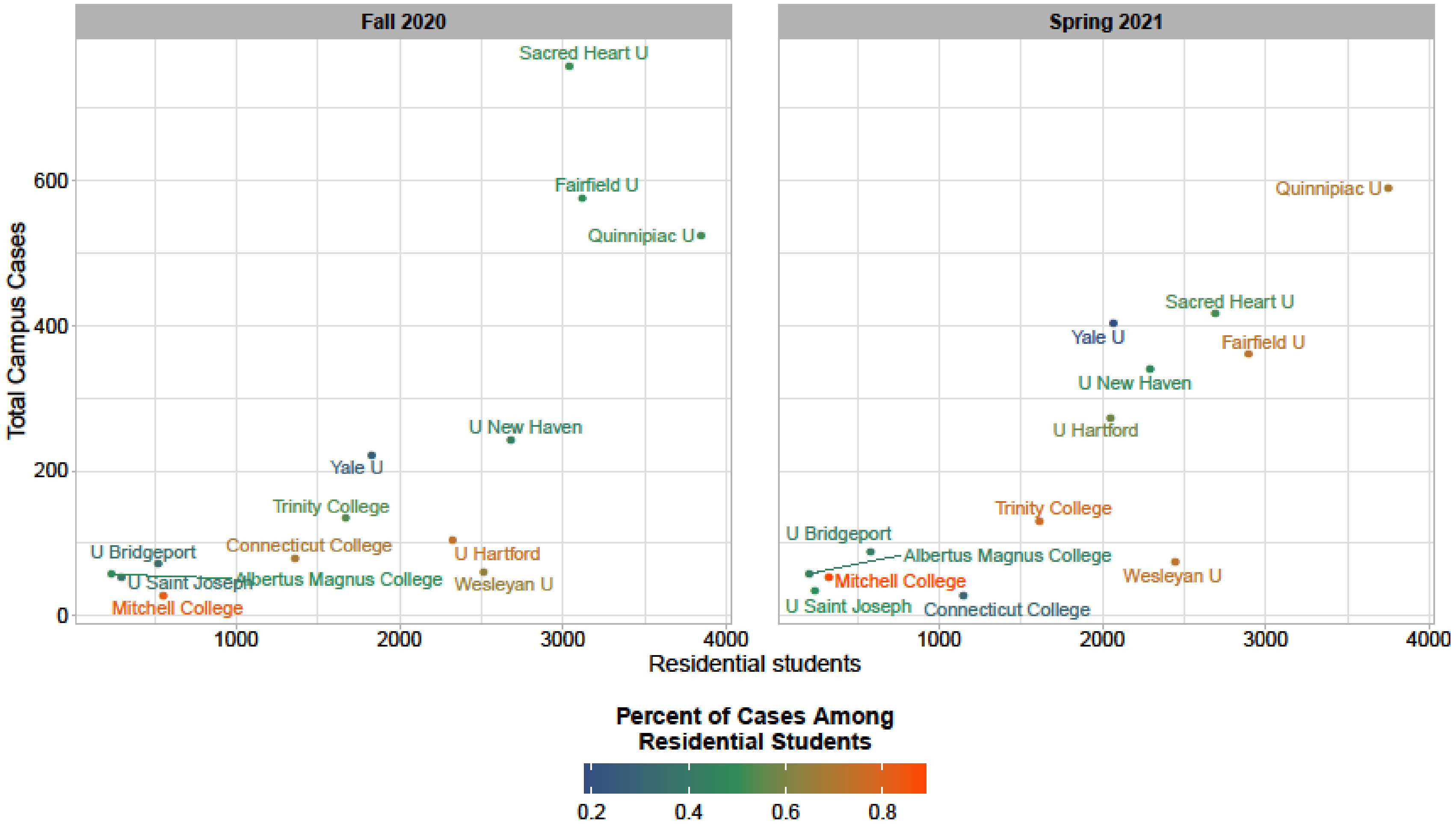

© 2021 Schultes O et al. *JAMA Network Open*.

eTable 1. University Characteristics, Residential Enrollment, and COVID-19 Plans and Policies for Residential Students During the 2020-2021 Academic Year

Bacc. = Baccalaureate; Quar. = quarantine

Institution	Institution Type	Highest Degree	Residential Enrollment		Repopulation		Monitoring		Sources
			Fall	Spring	Fall	Spring	Fall	Spring	
Albertus Magnus College	Private (Catholic)	Masters	235	203	Test <7 days prior, day of arrival; No quar. data	Test <7 days prior, day of arrival; 7 day quar.	5-10% tested weekly; Daily health check	All tested weekly through Feb, then 25%; Daily health check	Gstalder,[6] 2021; Barr,[7] 2020; Heitz,[8] 2021
Central Connecticut State University	Public	Masters	992	743	Test <14 days prior; 14 day quar. from high-risk areas	Test <7 days prior*, day of arrival, 5 days after; 7 day quar.	5-10% tested weekly; Daily health check	All tested weekly through Feb, then 25%; Daily health check	Cintorino,[9] 2020; Cintorino, 2020[10]
Connecticut College	Private	Bacc.	1358	1150	Test <7 days prior, day of arrival; 14 day quar. before/after arrival	Test <7 days prior; No quar. data	All tested 2x weekly; Daily health check	All tested 2x weekly; Daily health check	Arcelus,[11] 2020; Arcelus,[12] 2020; "Campus Life",[13] 2021; COVID-19 Response Team,[14] 2021
Eastern Connecticut State University	Public	Masters	1830	1579	Test <14 days prior; 14 day quar. from high-risk areas	Test <14 days prior, day of arrival, 5 days after; 7 day quar.	5-10% tested weekly; No daily health check	All tested weekly through Feb; Daily health check for first 7 days	DeLisa,[15] 2020; DeLisa,[16] 2020; "Housing and Student Life",[17] 2021
Fairfield University	Private (Catholic)	Masters	3119	2894	Test <14 days prior; 14 day quar. from high-risk areas	Test 5 days prior, 2-5 days after arrival; 7-18 day quar.	5-10% tested weekly; Daily health check	All tested weekly; Daily health check	Lawlor,[18] 2020; "Return to Campus",[19] 2020; Donoghue,[20] 2021

© 2021 Schultes O et al. *JAMA Network Open*.

Mitchell College	Private	Bacc.	553	325	Test <5 days prior; No quar. data	Test <3 days prior; 15-20 day quar.	5% tested weekly; Daily health check	All tested weekly; Daily health check	Wright,[21] 2020; "MiniMester",[22] 2021; "Returning to Campus",[23] 2020
Quinnipiac University	Private	Masters	3845	3750	Test <7 days prior, 6-12 days after; 14 day quar. from high-risk areas	Test 10 days prior, day of arrival; 11 day quar.	15% tested weekly; Daily health check	All tested weekly through Feb, then 20%; Daily health check	Zemba,[24] 2020; Drucker,[25] 2021; "8 new things",[26] 2021
Sacred Heart University	Private (Catholic)	Masters	3037	2690	Test <14 days prior; 14 day quar. from high-risk areas	Test <7 days prior, day of arrival; 14 day quar.	5-10% tested weekly; Daily health check	All tested weekly through Feb; Daily health check	MacNamara,[27] 2020; "Community Updates",[28] 2021
Southern Connecticut State University	Public	Masters	1433	1042	Test <14 days prior; 14 day quar. from high-risk areas	Test <14 days prior, day of arrival; 7 day quar.	5-10% tested weekly; Daily health check for first 7 days	All tested first week, then 5-10% weekly; Daily health check for first 7 days	Richardson,[29] 2020; Richardson,[30] 2021
Trinity College	Private	Bacc.	1670	1614	Test <5 days prior; 14 day quar. before or after*	Test <7 days prior, day of arrival; >7 day quar.	All tested 2x weekly for first 3 weeks, then weekly; Daily health check	All tested 2x weekly; Daily health check	Berger-Sweeney,[31] 2020; DiChristina,[32] 2020; DiChristina,[33] 2021; "CoVerified",[34] 2021
University of Bridgeport	Private	Masters	520	580	Test <14 days prior; 14 day quar. from high-risk areas	Test <5 days prior, 6-10 days after initial test; No quar. data	5-10% tested weekly; Daily health check	All tested weekly; Daily health check	Schmidt,[35] 2020; "FAQ",[36] 2021; Sanders,[37] 2021
University of Connecticut	Public	Doctoral	4603	4492	Test day of arrival; 14 day quar.	Test 5-7 days prior, day of arrival; 14 day quar.	5-10% tested weekly; Daily health check for first 7 days	All tested weekly through Feb; No daily health check	Jordan,[38] 2020; Jordan,[39] 2021

© 2021 Schultes O et al. *JAMA Network Open*.

University of Hartford	Private	Doctoral	2325	2050	Test <14 days prior; 14 day quar. from high-risk areas	Test <7 days prior, day of arrival; 8-11 day quar.	5-10% tested weekly; Daily health check	All tested weekly; Daily health check	Nicklin,[40] 2020; "Testing for COVID-19",[41] 2021; Office of Marketing and Communication,[42] 2021
University of New Haven	Private	Masters	2679	2289	Test 5 days prior; 14 day quar. from high-risk areas	Test 5 days prior, within 9 days of arrival; 7-9 day quar.	5% tested weekly; Daily health check	All tested weekly; Daily health check	McGee,[43] 2020; McGee,[44] 2021; McGee,[45] 2021
University of Saint Joseph	Private (Catholic)	Masters	296	239	Test <14 days prior; 14 day quar. from high-risk areas	No data available	5-10% tested weekly; Daily health check	No data available	Free,[46] 2020; DiSalvo,[47] 2021
Wesleyan University	Private	Bacc.	2516	2446	Test <3 days prior*; 7-14 day quar.	Test <7 days prior, day of arrival; 14 day quar.	All tested 2x weekly; No daily health check	All tested 2x weekly; No daily health check	Culliton,[48] 2020; "All About Testing",[49] 2021; Whaley,[50] 2020; "Safety Guidelines",[51] 2021; "Prepare for Arrival", 2021[52]
Western Connecticut State University	Public	Masters	805	686	Test <14 days prior; 14 day quar. from high-risk areas	Test <14 days prior; No quar. data	5-10% tested weekly; Daily health check for first 7 days	25% tested weekly; Daily health check for first 7 days	Koukopoulos,[53] 2020; Koukopoulos,[54] 2020
Yale University	Private	Doctoral	1829	2065	Test <14 days prior, day of arrival; 14 day quar.	Test <14 days prior, day of arrival; 8-10 day quar.	All tested 2x weekly; Daily health check	All tested 2x weekly; Daily health check	Spangler,[55] 2020; Boyd,[56] 2020; Chen,[57] 2021; "Yale Community Compact",[58] 2021

*recommended policy

© 2021 Schultes O et al. *JAMA Network Open*.

eTable 2. Linear Regression Estimates and 95% Confidence Intervals Describing the Negative Association Between Residential Test Rate (per Student per Week) and Residential Case Rate (per Student per Week), Adjusted for Town Case Rate per Person

	Estimate	2.5%	97.5%
Intercept	0.00411	0.00211	0.00611
Residential test rate	-0.00141	-0.00281	-0.00001
Town case rate	0.43244	-0.50911	1.37399

© 2021 Schultes O et al. *JAMA Network Open*.

eTable 3. Linear Regression Estimates and 95% Confidence Intervals Describing the Association Between Residential Case Rate (per Student per Week) and Town Case Rate (per Person per Week)

The association is estimated to be positive during fall 2020, and not significantly different from zero during spring 2021.

	**Estimate**	**2.5%**	**97.5%**
Intercept	0.00182	0.00169	0.00195
Fall 2020 residential case rate	0.08446	0.06065	0.10827
Spring 2021 residential case rate	0.01871	-0.00228	0.03970

© 2021 Schultes O et al. *JAMA Network Open*.

eTable 4. Association Between Tests per Student per Week and Cases per Student per Week, by Test Frequency

Tests per student per week	Estimate	2.50%	97.50%
(0,0.25]	-0.00062	-0.00195	0.00070
(0.25,0.5]	0.00145	-0.00012	0.00303
(0.5,0.75]	0.00279	0.00070	0.00489
(0.75,1]	0.00327	0.00187	0.00466
(1,1.25]	0.00501	0.00288	0.00713
(1.25,1.5]	0.00023	-0.00319	0.00365
(1.5,1.75]	-0.00069	-0.00326	0.00188
(1.75,2]	0.00122	-0.00066	0.00311
(2,∞]	0.00042	-0.00296	0.00381
town case rate	1.40494	1.00252	1.80736

© 2021 Schultes O et al. *JAMA Network Open*.

eReferences

1. Lamont N. Executive Order No. 7III: Protection of Public Health and Safety During COVID-19 Pandemic and Response – Mandatory Self-Quarantine of Travelers Arriving from States with High COVID-19 Infection Rates and Extension of Certain Deadlines Applicable to the Department of Motor Vehicles. State of Connecticut; 2020.
2. Ojakian M. Update #10 to the Higher Education Report: Recommendations for Reopening Undergraduate Colleges and Universities. *Guidance Related to Students from Affected States*: Higher Education Reopen Connecticut Subcommittee; 2020.
3. Connecticut Department of Public Health. Interim Guidance for the Reopening of Higher Education Campuses with On-Campus Residential Populations for the Spring 2021 Semester (01-08-2021). 2021.
4. Connecticut Department of Public Health. Interim Guidance for Surveillance Testing on Higher Education Campuses with On-Campus Residential Populations for the Spring 2021 Semester February 25, 2021. 2021.
5. Connecticut Department of Public Health. Interim Guidance for Surveillance Testing on Higher Education Campuses with On-Campus Residential Populations for the Spring 2021 Semester March 26, 2021. 2021.
6. Gstalder S. ALBERTUS MAGNUS COLLEGE COVID-19 SPRING 2021 PLANS. Albertus Magnus College; 2021.
7. Barr S. In Historic 95th Anniversary Year, Vigilance Pays Off As Albertus Magnus College Reaches Thanksgiving Break. Albertus Magnus College; 2020.
8. Heitz ET, Magenheimer E. Albertus Magnus College; 2020.
9. Cintorino S. CCSU Blueprint: Plans for a Successful Fall 2020 Reopening. Central Connecticut State University; 2020.
10. Cintorino S. CCSU Blueprint: Plans for a Successful Spring 2021 Reopening. Central Connecticut State University; 2020.
11. Arcelus V, McKnight J, Singer J. Important Conn Fall 2020 Information (pre-arrival requirements, campus testing andmove-in). Connecticut College; 2020.
12. Arcelus V, McKnight J, Singer J. Important Information for Spring 2021 Return. Connecticut College; 2020.
13. Campus Life in Spring 2021. 2021; https://www.conncoll.edu/campus-life/student-health-services/coronavirus/campus-life-with-covid-19/. Accessed May 17, 2021.
14. COVID-19 Response Team. Returning to Conn, Frequently Asked Questions. 2021; https://www.conncoll.edu/campus-life/student-health-services/coronavirus/returning-to-conn-faqs/. Accessed July 23, 2021.
15. DeLisa K, Rose-Zak S. Eastern Connecticut State University Reopening Plan, Fall 2020. Eastern Connecticut State University; 2020.
16. DeLisa K, Rose-Zak S. Eastern Connecticut State University Reopening Plan, Spring 2021. Eastern Connecticut State University; 2020.
17. Housing and Student Life. 2021; https://www.easternct.edu/reopening/student-support/housing.html. Accessed July 22, 2021.
18. Lawlor K. Reopening Plans for Undergraduate Residential Colleges and Universities in Phase 3 - Name of Institution: Fairfield University. Fairfield University; 2020.

© 2021 Schultes O et al. *JAMA Network Open*.

19. Plans for Fairfield's Spring 2021 Return to Campus. 2020; https://www.fairfield.edu/news/archive/2021/january/spring-reopening-return-to-campus.html?utm_source=watson&utm_medium=social&utm_campaign=mc&utm_term=spring-2021&utm_content=fairfield-university-reopening. Accessed June 11, 2021.
20. Donoghue K. A Message from the Vice President for Student Life. Fairfield University; 2021.
21. Wright C. Mitchell_College_State_plan_Phase3.pdf. Mitchell College; 2020.
22. Spring MiniMester Courses: May + June. 2021; https://mitchell.edu/wp-content/uploads/2021/05/May-June-MiniMester-2021-to-REGISTERED-CURRENT-students.pdf. Accessed June 14, 2021, 2021.
23. Information about Returning to Campus this Spring! : Mitchell College; 2020.
24. Zemba B. Quinnipiac University Reopening Plan Phase 3 Fall 2020. Quinnipiac University; 2020.
25. Drucker M. Return to Campus Guide for Students Spring 2021. Quinnipiac University; 2021.
26. 8 new things you need to know for the spring. 2021; https://www.qu.edu/quinnipiac-today/8-new-things-you-need-to-know-for-the-spring-2021-01-12/. Accessed July 22, 2021.
27. MacNamara G. Sacred Heart University Undergraduate Residential Phase 3 Reopening Campus Plan Fall 2020. Sacred Heart University; 2020.
28. Community Updates. Sacred Heart University; 2021.
29. Richardson A. Reopening Plans for Colleges and Universities in Phase 3 July 28, 2020. Southern Connecticut State University; 2020.
30. Richardson A. Reopening Plans for Colleges and Universities in Phase 3 January 2021. Southern Connecticut State University; 2021.
31. Berger-Sweeney J, Rojas J. Trinity College Health and Safety Plan for Reopening. Trinity College; 2020.
32. DiChristina J, Goodman J. Update on PCR COVID 19 Testing and State of Connecticut Travel Advisory. Trinity College; 2020.
33. DiChristina J, Rojas J. Preparing for the spring semester at Trinity College. Trinity College; 2021.
34. CoVerified App - Reopening. 2021; https://www.trincoll.edu/reopening/coverified-app/. Accessed June 14, 2021.
35. Schmidt R. University of Bridgeport Re-opening Plan Phase 3. University of Bridgeport; 2020.
36. Frequently Asked Questions. 2021; https://www.bridgeport.edu/covid-19/faq. Accessed June 14, 2021.
37. Sanders C. Important Move-In Information. University of Bridgeport; 2021.
38. Jordan S. Reopen Phase 3 Fall Semester Commencing on or after 14 August 2020. University of Connecticut; 2020.
39. Jordan S. Reopen Plan Spring Semester Commencing 16 January 2021. University of Connecticut; 2021.
40. Nicklin J. Reopening Plan for the University of Hartford in Phase 3 - Fall 2020. University of Hartford; 2020.

© 2021 Schultes O et al. *JAMA Network Open*.

41. Testing for COVID-19. 2021; https://www.hartford.edu/healthy-hawks/health-safety-guidelines/covid-testing.aspx#accordion-group-1-section-1-label. Accessed June 11, 2021.
42. Office of Marketing and Communication. Move-In Updates. University of Hartford; 2021.
43. McGee S. Reopening Plan - Fall 2020. University of New Haven; 2020.
44. McGee S. University Response to COVID-19: Important Notifications and FAQ. 2021; https://www.newhaven.edu/covid19/notifications-and-faq.php. Accessed June 11, 2021.
45. McGee S. The Phased Plan for Return to Campus - Spring 2021: Phase 3. University of New Haven.
46. Free R. Reopening Plans for Colleges and Universities in Phase 3 - Fall 2020. University of Saint Joseph; 2020.
47. DiSalvo E. Colleges Start Spring Semester with Souped up Testing. 2021; https://we-ha.com/colleges-start-spring-semester-with-souped-up-testing/. Accessed July 23, 2021.
48. Culliton R. Reopening Plans for Undergraduate Residential Colleges and Universities in Phase 3. Wesleyan University; 2020.
49. All About Testing. Wesleyan University.
50. Whaley M, Roth M. Important Information for Fall Semester. Wesleyan University; 2020.
51. Safety Guidelines. Wesleyan University.
52. Prepare for Arrival. 2021; https://www.wesleyan.edu/reactivatingcampus/prepare-arrival.html. Accessed July 22, 2021.
53. Koukopoulos P. Reopening Plans for Colleges and Universities in Phase 3 - Fall 2020. Western Connecticut State University; 2020.
54. Koukopoulos P. Reopening Plans for Colleges and Universities in Phase 3 - Spring 2021. Western Connecticut State University; 2020.
55. Spangler S, Loucks N. Yale University “Reopen Connecticut” Phase 3 Plan. Yale University; 2020.
56. Boyd M. Spring 2021 housing and move-in information (December 8). Yale University; 2020.
57. Chen C. Students: Pre-Arrival Testing for Spring 2021. Yale University; 2021.
58. Yale Community Compact. Yale University; 2021.

© 2021 Schultes O et al. *JAMA Network Open*.
